# Should Expert Surgeon Guidance Be Given Remotely? Comment on “User Experience in Remote Surgical Consultation: Survey Study of User Acceptance and Satisfaction in Real-Time Use of a Telemedicine Service”

**DOI:** 10.2196/36681

**Published:** 2022-06-29

**Authors:** Daniella Soussi, Chiara Jade Vedi

**Affiliations:** 1 School of Medicine Imperial College London London United Kingdom

**Keywords:** telemedicine, user experience, satisfaction, technology acceptance, usability, perioperative, surgery, consultation, surgeons, performance, evaluation, tele-guidance, telehealth, telemedicine implementation, telementoring, surgical consultation, usefulness

We recently read with great interest the article “User Experience in Remote Surgical Consultation: Survey Study of User Acceptance and Satisfaction in Real-Time Use of a Telemedicine Service” by Aminoff et al [1], where a real-time telemedicine service was used for intraoperative surgical consultation assistance during endoscopic retrograde cholangiopancreatography procedures. The authors investigated the surgeons’ preprocedure expectations of how the service would aid operative performance and patient outcomes, and their satisfaction after use of the service. We commend the authors for their work; however, we would like to highlight some caveats in their research.

Although this study presents interim results, we are concerned that the perceived future use of the service may be overestimated. Expert surgeons had to book out their time in advance to participate, and when the sample was asked about the perceived demand for teleguidance, 80% said they believed there would be no demand for it or were unsure of the demand [1]. This coupled with the fact that sites were encouraged to use the service due to being part of a trial makes it unclear as to whether the telemedicine service would be used in practice.

Moreover, technical issues experienced in virtual services can prohibit their successful implementation [2]. Technical issues were apparent in 24% of cases, and problems in audio and video connection and fluoroscopy transfer could lead to incorrect surgical decision-making guidance from the remote expert. Even though some of these issues were resolved either through connection restarting or calling medical technicians, these unnecessary delays increase patients’ time under anesthesia, which is known to increase perioperative complications such as infection [3]. Indeed, this could explain the 4 (2.8%) cases that had postoperative complications; although, we cannot confirm this as a causal inference [1]. Furthermore, due to the limitations in the content being transferred at any one time via the service and the lack of 3D visualization, we caution against the use of remote expert guidance in particularly difficult surgical decision-making scenarios.

Although novice endoscopic retrograde cholangiopancreatographists demonstrated an increase in their level of expertise during the study period, we argue that this intervention may be time-inefficient, with 81.1% of the procedure time being spent on receiving guidance [1]. Furthermore, no follow-up data is available to predict whether skills would be maintained or regress if tele-guidance were not implemented, which casts doubt on the service’s training value.

Previous work has found that *telestration* features can reduce the time of mentoring sessions [4]. However, due to the user-unfriendly design of this particular *telestration* tool, this benefit was unlikely. Therefore, we suggest the development of a more tailored design and function to create more time for other clinical duties consulting surgeons may have.

Finally, to determine the true value of this service in clinical practice, we recommend a study investigating the acquisition of both technical and nontechnical skills for on-site expert-guided surgeries compared to remote surgical consultation.

In conclusion, we commend the authors for their innovative surgical guidance service and suggest that the aforementioned are considered when formally implementing the service in the clinical setting.
